# *Ginseng Radix et Rhizoma* enhanced the effect of metoprolol in chronic heart failure by inhibiting autophagy in male C57BL/6J mice

**DOI:** 10.1371/journal.pone.0301875

**Published:** 2024-08-14

**Authors:** Niu Zi-chang, Han Xiao-ling, Jin Qi, Liu Ting, Ouyang Ming-hui, Mao Hao-ping

**Affiliations:** 1 Key Laboratory of Pharmacology of Traditional Chinese Medical Formulea, Ministry of Education, Tianjin University of Traditional Chinese Medicine, Tianjin, People’s Republic of China; 2 First Teaching Hospital of Tianjin University of Traditional Chinese Medicine, Tianjin, People’s Republic of China; Chosun University, REPUBLIC OF KOREA

## Abstract

**Background:**

*Ginseng Radix et Rhizoma* (GS) is frequently used as an adjuvant therapy for patients with heart failure (HF). Metoprolol is widely used in patients with HF. However, there is no report on the combined effects of GS and metoprolol in patients with HF.

**Objective:**

This study investigated the combined effects of GS and metoprolol in male C57BL/6J mice with HF and the underlying mechanisms.

**Materials and methods:**

We utilized a mouse myocardial HF model to measure the serum levels of creatine kinase (CK) and creatine kinase-MB form (CK-MB) using an automated biochemical analyzer. Lactate dehydrogenase (LDH) and cardiac troponin (cTnT) levels were determined using enzyme-linked immunosorbent assays. Autophagy of myocardial cells was evaluated using transmission electron microscopy, and changes in signal pathway proteins related to autophagy were analyzed by Western blotting.

**Results:**

GS combined with metoprolol improved heart function, reduced heart damage, and decreased serum levels of CK, CK-MB, LDH, and cTnT. The combination of GS and metoprolol decreased autophagy in myocardial cells by reducing the levels of autophagy-related proteins (LC3, p62, Beclin1, and Atg5) and increasing the ratios of p-PI3K/PI3K, p-Akt/Akt, and p-mTOR/mTOR.

**Conclusion:**

GS enhanced the anti-heart failure effect of metoprolol. Its mechanism of action might be related to the inhibition of autophagy mediated by the activation of the PI3K/Akt/mTOR pathway.

## Introduction

Heart failure (HF), a complex syndrome characterized by heart dysfunction, is typically caused by a reduced ability to pump blood. It is normally caused by arrhythmia, cardiomyopathy, hypertension, coronary heart disease, etc. It is a global public health issue that impacts over 64 million individuals [1]. Heart failure is becoming one of the leading causes of death, attributing to a significant increase in prevalence [2]. Therefore, the development of efficient HF therapies is of great significance in reducing the incidence and mortality of HF.

Significant advancements in HF treatment strategies have been made in recent decades. Numerous medications, such as beta-adrenergic receptor blockers (β-blockers), angiotensin-converting enzyme inhibitors (ACEIs), angiotensin receptor blockers (ARBs), angiotensin receptor–neprilysin inhibitors (ARNIs), mineralocorticoid receptor antagonists (MRAs), and sodium-glucose cotransporter 2 (SGLT2) inhibitors, have been shown to improve cardiac function, quality of life, and survival rates in individuals with HF [3, 4]. Among these medications, beta-blockers are essential and widely used in HF. Several randomized controlled trials (RCTs) have shown that beta-blockers decreased hospitalization, sudden cardiac death, and all-cause cardiovascular mortality in patients with HF [5]. Consequently, beta-blockers should be prescribed to all patients with HF who are not restricted or intolerant to them.

*Panax ginseng C*. *A*. *Mey*., known as the “king of herbs,” is primarily cultivated in China, South Korea, and the United States. Historically, the medicinal properties of the plant have been traditionally attributed to its roots (*Ginseng Radix et Rhizoma*, GS for short). Applications have expanded to the leaves, stems, fruits, and flowers nowadays [6]. The medicinal use of GS spans approximately 2000 years, with the initial documentation in the “Shennong Materia Medica Classic.” In China, GS is included in the “Catalogue Management Regulations for Substances that are Traditional Food and Chinese Medicinal Materials.” The United States Pharmacopeial Convention officially recognizes GS as a dietary supplement. GS is characterized by many active ingredients, including ginsenosides, polysaccharides, polyethylene, amino acids, and phenolic compounds [7]. Among these components, ginsenoside is the most notable, boasting the highest concentration and the most extensive research on its efficacy [8]. Ginsenoside is frequently used as a standard for identification and content determination in pharmacopeias in China and Japan, highlighting the significant role in the therapeutic value of GS. Recent research indicated that GS and preparations possessed diverse pharmacological activities, including anti-cancer, immune regulation, anti-inflammatory, anti-allergy, anti-atherosclerosis, anti-hypertensive, anti-diabetic, anti-stress, anti-oxidative, and anti-apoptotic properties [9, 10]. Due to the numerous benefits, this herb is used to treat various diseases and plays a significant role in promoting health. GS preparations, including oral liquids, tablets, and injections, have been officially registered and marketed. These preparations have shown favorable clinical efficacy in treating cardiovascular diseases, regulating immunity, improving glucose and lipid metabolism, relieving fatigue, adjuvant treatment of tumors, alleviating inflammation and pain, and delaying aging [11].

Zhang Xichun, in his book “Medical Zhongzhong Canxi Lu,” exemplified the integration of Chinese and Western medicinal practices since the Qing Dynasty with the “Gypsum Aspirin Soup.” In China, a combination of Eastern and Western medicines is widely applied in treating cardiovascular diseases. Reports indicated that GS had a synergistic effect when used with other drugs. The combination of GS and tetramethylpyrazine phosphate significantly improved the survival rate and heart function in transgenic mice with cardiomyopathy compared to the group that received only tetramethylpyrazine phosphate. It enhanced superoxide dismutase (SOD) activity, reduced pathological damage to myocardial cells and myocardium, decreased infarct/ischemic area, and improved heart function, indicating its synergistic effect in the treatment of heart diseases [12]. Furthermore, a work underscored the substantial amplification of cisplatin’s anticancer potency and the mitigation of its harmful side effects when administered concomitantly with ginsenosides [13]. Besides, the combination of ginsenoside Rb1 ameliorated cardiotoxicity triggered by aconitine which improved myocardial cell viability and synergistically enhanced efficacy in treating acute heart failure [14].

The clinical application of beta-receptor blockers, such as metoprolol (Met), is extensive. More and more studies focused on the combination of Met with other medicines [15, 16]. However, the effect and underlying mechanisms of combining GS and Met in treating heart failure have not been elucidated. Therefore, we conducted this study to investigate the impact and potential mechanism of combining GS with Met, a beta-receptor blocker, in treating HF in male C57BL/6J mice.

## Materials and methods

### Drugs and reagents

Metoprolol tartrate tablets (H32025391) were obtained from MedChemExpress. (Shanghai, China). GS used in the present work was extracted from *Ginseng Radix et Rhizoma* purchased from Beijing Tongrentang Pharmaceutical Co., Ltd (Beijing, China). Standards of ginsenoside Re, Ginsenoside Rg1, Ginsenoside Rf1, and Ginsenoside Rb1 were purchased from Chengdu Desite Biotechnology Co., Ltd (purity > 99%, Chengdu, China) Hematoxylin-eosin (HE, catalog number C0105S) and Masson trichrome (C0189S) were purchased from beyotime (Shanghai, China). Enzyme-linked immunosorbent assay (ELISA) kits for mouse cardiac troponin (cTnT, catalog number was E-EL-M1801) were purchased from Elabscience (China). ELISA kits for the serum biochemical indicators lactate dehydrogenase (LDH), creatine kinase (CK), and creatine kinase, MB form (CK-MB) (catalog number was BS-300) were purchased from Shenzhen Mindray Biomedical Electronic Co., Ltd (Shenzhen, China). Rabbit monoclonal anti-LC3 (ab192890), anti-Beclin1 (ab207612), anti-Atg5 (ab108327), anti-PI3K (ab191606), anti-Akt (ab179463), anti-p-Akt (ab192623), and anti-mTOR (ab134903) were purchased from Abcam (Cambridge, UK). Anti-p-PI3K (17366), anti-p-mTOR (5536), and rabbit monoclonal anti-p62 (5114) was purchased from CST (Boston, USA). Goat anti-rabbit IgG were purchased from Beijing Zhongshan Jinqiao Biotechnology Co., Ltd.

### Preparation of GS for animal experiments

The voucher specimen of *Ginseng Radix et Rhizoma* was deposited at the Tianjin State Key Laboratory of Modern Chinese Medicine of the Tianjin University of Traditional Chinese Medicine. GS was extracted as follows: 500 g *Ginseng Radix et Rhizoma* was extracted with 60% ethanol (5 L) in a reflux apparatus two times, 2h each time. After refluxing, the collected extracts were evaporated in a rotary evaporator, and the samples were lyophilized in a freeze-dryer. Finally, the extract yield of GS was 21.4% of crude herb. Before it was used, GS was dissolved in distilled water to prepare a stock solution containing 0.214 g extraction in 1mL (1 g crude herb/mL,), and then 1.3 mL stock solution was diluted into 10 mL GS solution using distilled water freshly. *Ginseng Radix et Rhizoma* was refluxed with 60% ethanol twice. Then it was concentrated and dissolved in distilled water to prepare a stock solution (1 g crude herb/mL). Before it was used, 1.3 mL stock solution was diluted into 13 mL GS solution using distilled water.

### Ultra-performance liquid chromatography analysis

A Waters Ultra Performance Liquid Chromatography (UPLC) system coupled with a PDA detector was used to perform the quantitative analysis of the target compounds. All separation was performed on an ACQUITY UPLC BEH C18 column (2.1×100 mm, 1.7 μm). The flow rate was set as 0.3 mL/min. The column temperature was 40°C. The mobile phase comprised (A) aqueous formic acid (0.1%, v/v) and (B) methanol. The following gradient elution program was used: 30–52% B at 0–4 min, 52–57% B at 4–6 min, 57–65% B at 6–7 min, 65–69% B at 7–8 min, 69–73% B at 8–9 min, 73–75% B at 7–10 min, 75–80% B at 10–11 min, 80–90% B at 11–13 min, and 90–30% B at 13–15 min. The post-run-time was held at 5 min. The scan wavelength was 203 nm and the injection volume was 4 μL. GS (50 mg/mL) was centrifuged and filtered through a 0.22 μm membrane. Ginsenoside Re, Ginsenoside Rg1, Ginsenoside Rf1, and Ginsenoside Rb1 (purity > 99%, purchased from the National Institute for the Control of Pharmaceutical and Biological Products (Beijing, China)) were dissolved in methanol to produce 1 mg/mL stock solutions, which were serially diluted to draw the calibration curve. All working solutions were stored at 4°C until use.

### Animals

Male 8-week-old C57BL/6J mice (n = 7–10 in each group, 20 ± 2 g) were purchased from Beijing Vital River Laboratory Animal Technology Co., Ltd. (Beijing, China, Certificate No.: SCXK Jing 2014–0004). They were housed under controlled temperature (23–26°C) and humidity (40–60%) conditions with a 12/12 h light/dark cycle. Animal health and behavior were monitored every 24 hours.

### Ethics statement

This study was carried out in strict accordance with the recommendations in the Guide for the Care and Use of Laboratory Animals of the National Institutes of Health. The protocol was approved by the Laboratory Animal Ethics Committee approved the protocol of Tianjin University of Traditional Chinese Medicine (Permit Number: TCM-LAEC2020086). All surgery was performed under isoflurane anesthesia, and all efforts were made to minimize suffering. No animals died or showed signs of discomfort, distress, or suffering before the experiment was finished. After 24h of the last administration, euthanasia was carried out by first anesthetizing the mice with isoflurane and then performing cervical dislocation.

### Left anterior descending coronary artery (LAD) ligation

Mice were anesthetized by inhalation of isoflurane. Then they were endotracheally intubated and maintained with ventilator-assisted breathing. The respiratory parameters were set so that the respiratory rate was 133 times/min, the tidal volume was 0.2 mL, and the total tidal volume per minute was 26 mL. Next, the chest was opened at the intercostal space between the third and fourth sternal ribs via a left thoracotomy. An 8–0 silk suture was used to ligate the proximal left anterior descending coronary artery (LAD) under the left auricle. After successful ligation, the apex of the heart turned white. After suturing the thorax closure, the mice were released and placed on an electric blanket. The surgical procedures were identical in sham control mice, except the left anterior descending coronary artery was not tied. The clinical use of metoprolol (Met) to treat heart failure is as follows: Initially, a dose of 6.25 mg Met is used 2–3 times a day. Then an increase of 6.25–12.5 mg should be administered every few days to a week. The maximum dose can be 200 mg daily. The dosage in mice was converted into the dosage in humans by the equation:

$$Dose\;in\;humans_{\left(70kg\;weight\right)}=dose\;in\;mice_{\left(20g\;weight\right)}*0.02\;kg*387.9.$$

In the equation, 387.9 is the ratio of body surface area between humans _(weight: 70kg)_ and mice _(weight: 20g)_ [17]. That means 6.25 mg/kg/day in mice equals 48.49 mg/day _(70 kg weight)_. That means that we used 6.25 mg/kg Met in mice a day which was approximately 50 mg/person/day in our present work. The normal dose of *Ginseng Radix et Rhizoma* is 3–9 g (for an adult weighing 70 kg) according to the Chinese Pharmacopoeia 2015. In some clinical situations, the dose of GS could be up to 55.2 g [18]. We converted the dosage of 10 g GS in humans into 1.3 g/kg in mice according to the above equation. The mice were randomly divided into the following seven groups: sham, model, 6.25 mg/kg Met, 1.3 g/kg GS, 2.6 g/kg GS, 6.25 mg/kg Met + 1.3 g/kg GS, and 6.25 mg/kg Met + 2.6 g/kg GS.

### Echocardiography

Left ventricular function was noninvasively assessed using an ultra-high-resolution small animal ultrasound imaging system (Vevo 2100, VisualSonics, Toronto, ON, Canada) with a 30-MHz transducer. The following parameters indicating cardiac function were measured with M-mode: left ventricular ejection fraction (EF%), left ventricular fractional shortening (FS%), left ventricular internal diameter at diastole (LVID,d), left ventricular internal diameter at systole (LVID,s), interventricular septum thickness at diastole (IVS,d) and interventricular septum thickness at systole (IVS,s).

### Histopathological examination

After being treated for 28 days, the mice were sacrificed under anesthesia. The heart was harvested, fixed in 4% paraformaldehyde solution for 48 hours, and embedded in paraffin. Next, 5-μm-thick sections were cut from each segment and stained with hematoxylin-eosin (HE) and Masson trichrome. We used a microscope (Zeiss, China) to observe and image the sections.

### Measurement of biochemical parameters

The mice in each group were treated for 28 days. Then blood samples were collected, and serum was separated by centrifugation at 3500 rpm for 15 min. Serum levels of CK, CK-MB, and LDH were determined using an automatic biochemical analyzer (Mindray, China). Serum levels of cTnT were measured using an ELISA kit.

### Western blot analysis

The protein levels of LC3, p62, Beclin1, Atg5, PI3K, p-PI3K, Akt, p-Akt, mTOR, and p-mTOR were determined using Western blot. Cold lysis buffer was used to extract the total protein from the hearts. After centrifugation, the protein concentration in the supernatants was determined with a BCA Protein Assay Kit (Thermo, USA). Equal amounts of protein were separated using SDS-PAGE and transferred to polyvinylidene difluoride membranes, which were washed with Tris-buffered saline (TBS), blocked with TBS containing 5% skim milk or bovine serum albumin, incubated overnight at 4°C with primary antibody in TBST, washed, incubated with secondary antibody, and washed again. Finally, the protein bands were visualized using a chemiluminescence reagent, and signals were recorded using a chemiluminescence instrument (Bio-Rad Laboratories Inc., Hercules, CA, USA). The signal intensities were quantified using ImageJ.

### Statistical analysis

Experimental data are presented as the mean ± SEM. Statistical analysis was performed with a normality test to ascertain whether the data distribution was normal or non-normal. Then a one-way ANOVA for multiple groups was carried out followed by Dunnette’s post hoc test for data with normal distribution. In the latter case, the Kruskal-Wallis test was used followed by Dunn’s multiple comparison. Values of p < 0.05 or p < 0.01 were considered to indicate a statistically significant difference.

## Results

### The contents of GS

**Fig 1:**
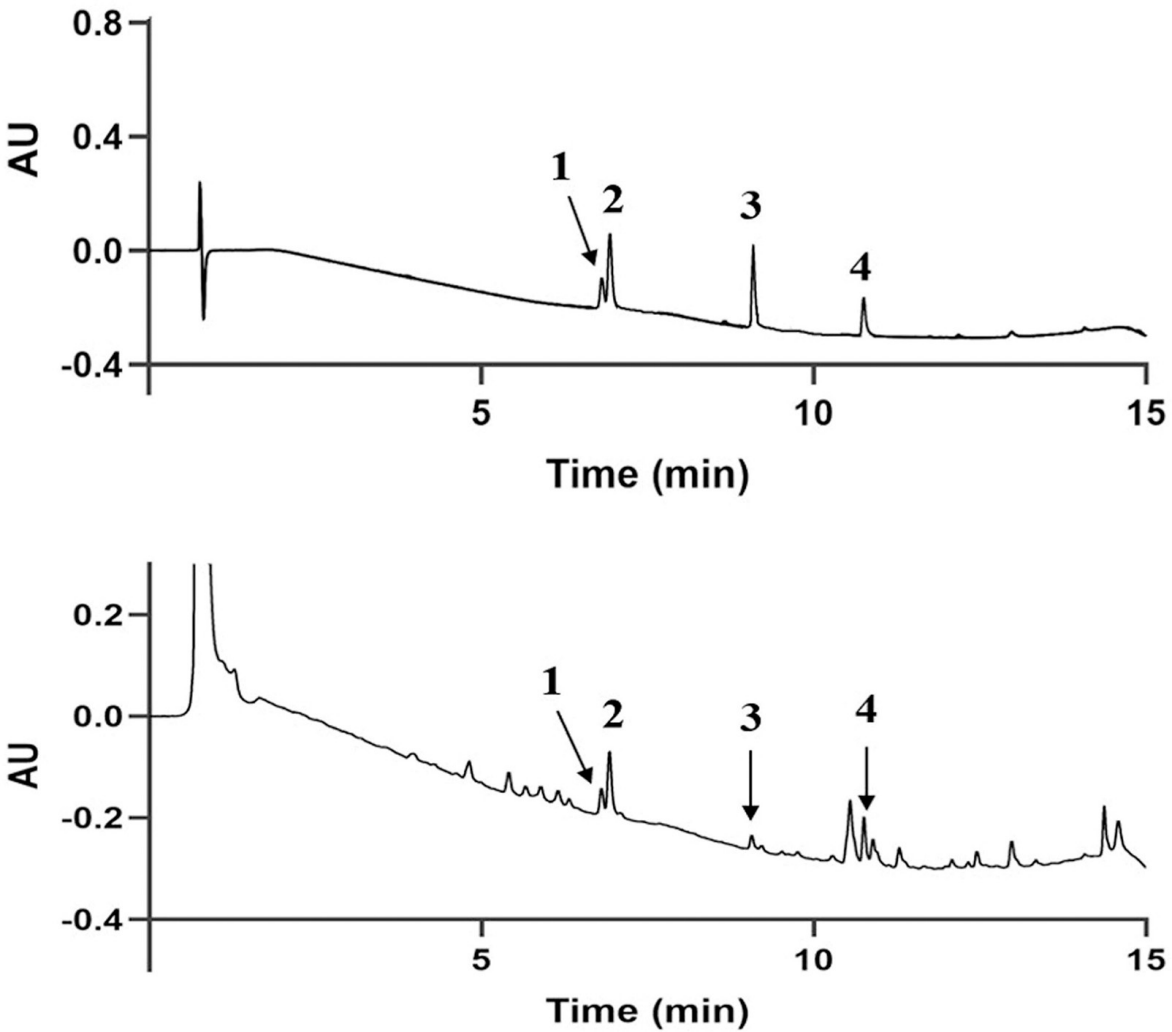
Typical chromatograms of (A) standard compounds and (B) samples. (1) Ginsenoside Re, (2) Ginsenoside Rg1, (3) Ginsenoside Rf1 and (4) Ginsenoside Rb1.

The contents of the GS extract were analyzed using UHPLC-PDA. Ginsenoside Re, Ginsenoside Rg1, Ginsenoside Rf1, and Ginsenoside Rb1 were detected and quantified. Representative chromatograms of the standards and GS are shown in **Fig 1**. The results showed that the GS contained 0.49 ± 0.01 mg/g Ginsenoside Re, 2.55 ± 0.03 mg/g Ginsenoside Rg1, 0.50 ± 0.03 mg/g Ginsenoside Rf1. and 2.70 ± 0.01 mg/g Ginsenoside Rb1.

### GS combined with metoprolol improved cardiac function

**Fig 2:**
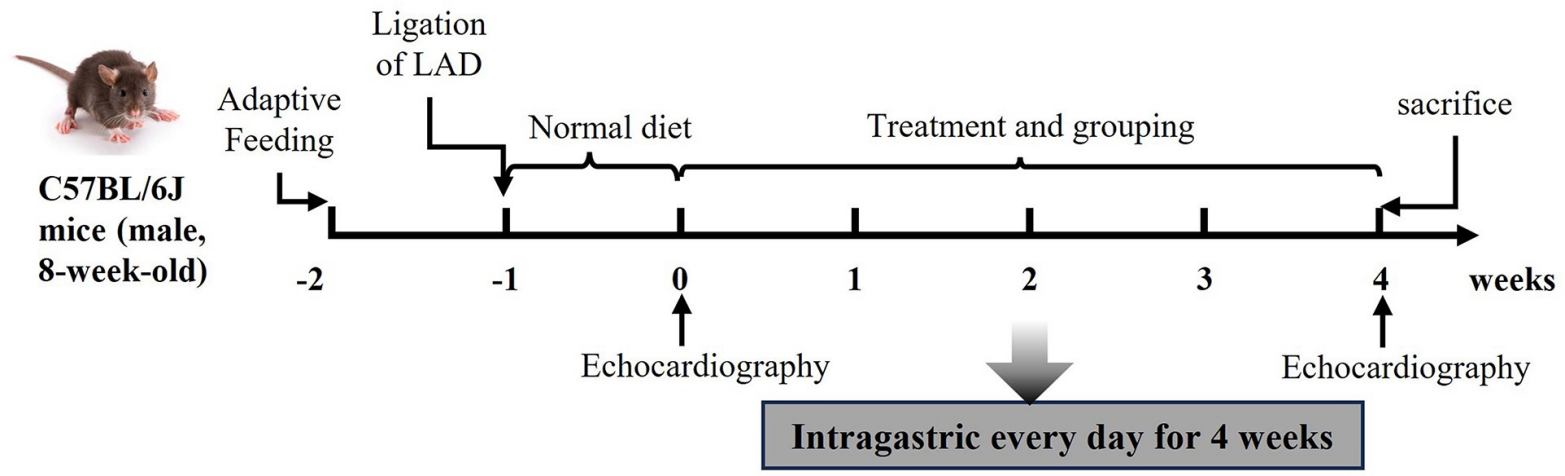
The schematic representation of experimental animal research design.

**Fig 3:**
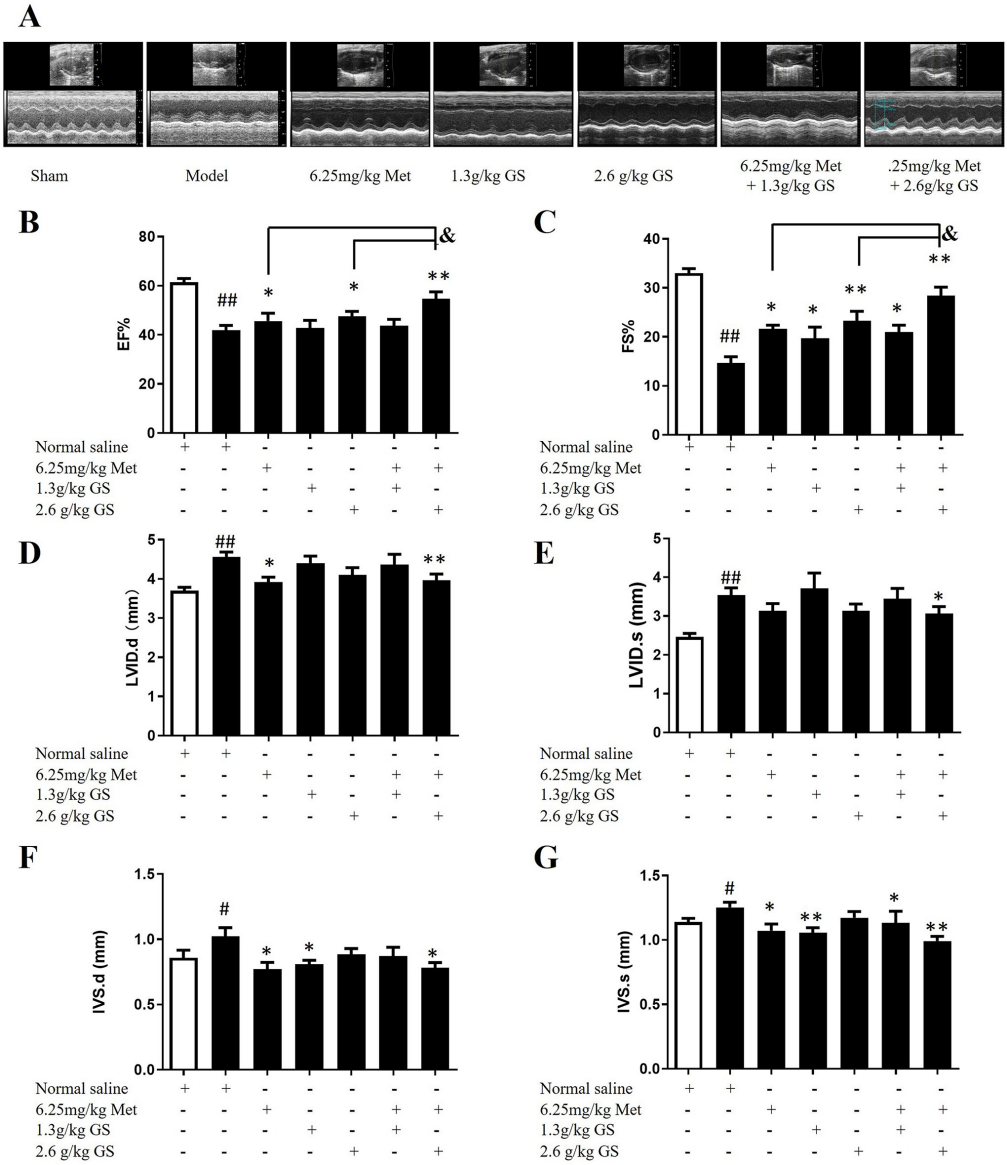
Typical echocardiograms (A), left ventricular ejection fraction (EF%) (B), left ventricular fractional shortening (FS%), left ventricular internal diameter at diastole (LVID,d) (D), left ventricular internal diameter at systole (LVID,s) (E), interventricular septum thickness at diastole (IVS,d) (F), and interventricular septum thickness at systole (IVS,s) (G) of heart failure mice treated with GS combined with metoprolol (Met). Data are represented as mean ± SEM (n = 7–10). #p < 0.05 or ##p < 0.01 vs. sham. *p < 0.05 or ** p < 0.01 vs. model. & p < 0.05 vs. 2.6g/kg GS or 6.25mg/kg Met.

Fig 2 shows the schematic representation of experimental animal research design. We used echocardiography to assess cardiac function in the mice (**Fig 3A**). Compared with the sham group, the EF% and FS% of the mice were significantly decreased in the model group (p < 0.01), suggesting the mice after ligation of LAD were suffering HF. A significant increase in EF% was recorded when mice were treated with 6.25 mg/kg Met only. Mice treated with Met at a dose of 6.25 mg/kg also showed a significant increase in FS% compared with the model (p < 0.05). Compared with the model, mice treated with 2.6 g/kg GS showed an elevation in EF% and FS% (p < 0.05 or p < 0.01). Furthermore, we found that the combination of 2.6 g/kg GS with Met significantly increased EF% and FS% compared with the Met and 2.6 g/kg GS groups (p < 0.05, **Fig 3B and 3C**). Model mice also showed increases in LVID,d and LVID,s (p < 0.01, **Fig 3D and 3E**), as well as increases in IVS,d and IVS,s (p < 0.05, **Fig 3F and 3G**). The combined use of 2.6 g/kg GS and 6.25 mg/kg Met significantly reversed the changes in the above factors compared with the model group (p < 0.01 or p < 0.05, **Fig 3D–3G**). The above results indicate that GS combined with Met could improve LAD ligation-induced injury in cardiac function.

### GS combined with Met reduced heart injury and serum levels of CK, CK-MB, LDH, and cTnT in mice with HF

**Fig 4:**
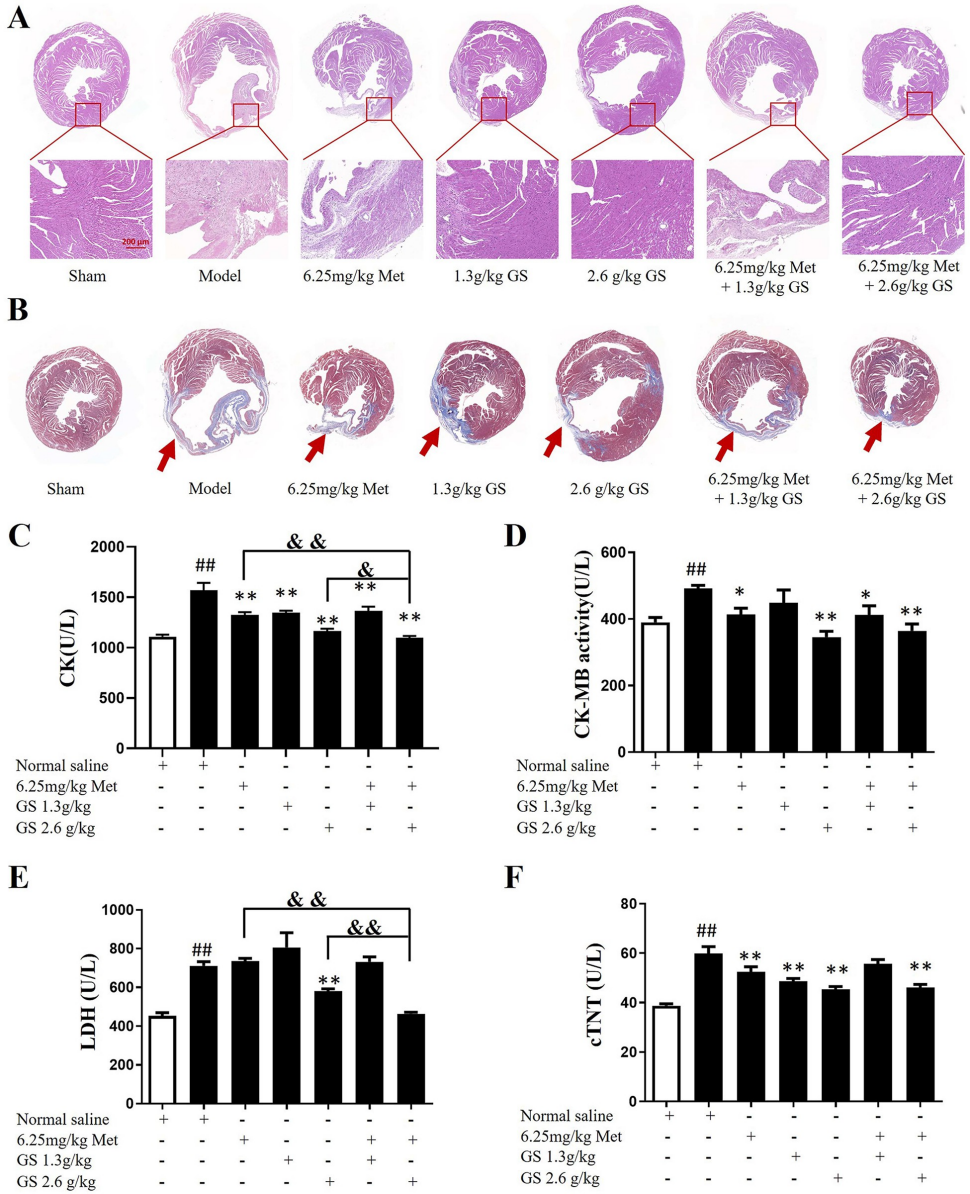
Effects of GS combined with metoprolol (Met) on myocardial morphology and serum levels of CK, CK-MB, LDH, and cTnT in heart failure mice. (A) Hematoxylin-eosin (HE) staining. (B) Masson trichrome staining. (C) Serum levels of creatine kinase (CK). (D) Creatine kinase, MB form (CK-MB). (E) Lactate dehydrogenase (LDH). (F) Cardiac troponin (cTnT). Data are represented as mean ± SEM (n = 7–10). ## p < 0.01 vs. sham. * p < 0.05 or ** p < 0.01 vs. model. & p < 0.05 or && p < 0.01 vs. 2.6g/kg GS or 6.25mg/kg Met.

The HE staining results showed that the cardiomyocytes in the sham group were neatly arranged, the intercellular space was uniform, and the structure was complete. In the model group, the myocardial cells were loosely arranged and disordered, the intercellular space was widened, and the myocardial stripes were broken or disappeared. Compared with the model group, cardiomyopathy was significantly reduced in the 2.6 g/kg GS group and the 6.25 mg/kg Met + 2.6 g/kg GS group (**Fig 4A**). Masson staining showed that the myocardial tissue (stained red) in the sham group was arranged orderly, and no collagen fibers appeared in the visual field. After LAD ligation, fibrosis and scarring appeared in the infarct border area of the myocardial tissue of the mice in each group, and the left ventricular wall (shown in red arrows) became thinner, especially in the model group. In addition, the remaining myocardial tissue in the model group was less, the distribution of myocardial cells was uneven, and the structure was severely damaged. Compared with the model group, the degree of myocardial fibrosis and the area of collagen fibers were significantly reduced in the 6.25 mg/kg Met + 2.6 g/kg GS treatment group (**Fig 4B**).

An automatic biochemical analyzer was used to determine the serum levels of CK, CK-MB, and LDH on the 28th day after administration. They are sensitive in myocardial tissue damage and were increased in the process of myocardial damage. As shown in **Fig 4C**, the serum CK content was significantly increased in the model group compared with the sham group (p < 0.01). Compared with the model group, the serum CK levels in the other groups were significantly decreased (p < 0.01). Compared with the 6.25 mg/kg Met group or the 2.6 g/kg GS group, the serum CK content in the 6.25 mg/kg Met + 2.6 g/kg GS group was significantly decreased (p < 0.01 or p < 0.05). As shown in **Fig 4D**, the serum CK-MB content in the model group was significantly increased compared with the sham group (p < 0.01). Compared with the model group, the serum levels of CK-MB were reduced in the 6.25 mg/kg Met, 2.6 g/kg GS, 6.25 mg/kg + Met1.3 g/kg GS, and 6.25 mg/kg Met + 2.6 g/kg GS groups (p < 0.05 or p < 0.01). As shown in **Fig 4E**, serum LDH levels were significantly increased in the model group compared with the sham group (p < 0.01). Serum LDH levels were significantly decreased in the 6.25 mg/kg Met + 2.6 g/kg GS group compared with the 2.6 g/kg GS group or the 6.25 mg/kg Met group (p < 0.01). The serum cTnT level in the model group was significantly increased compared with the sham group (p < 0.01) (**Fig 4F**). The cTnT levels in the 6.25 mg/kg Met, 1.3 g/kg GS, 2.6 g/kg GS, and 6.25 mg/kg Met + 2.6 g/kg GS groups were significantly decreased (p < 0.01).

### Effects of GS combined with Met on autophagy-related proteins (LC3, p62, Beclin1, and Atg5) in mice with HF

**Fig 5:**
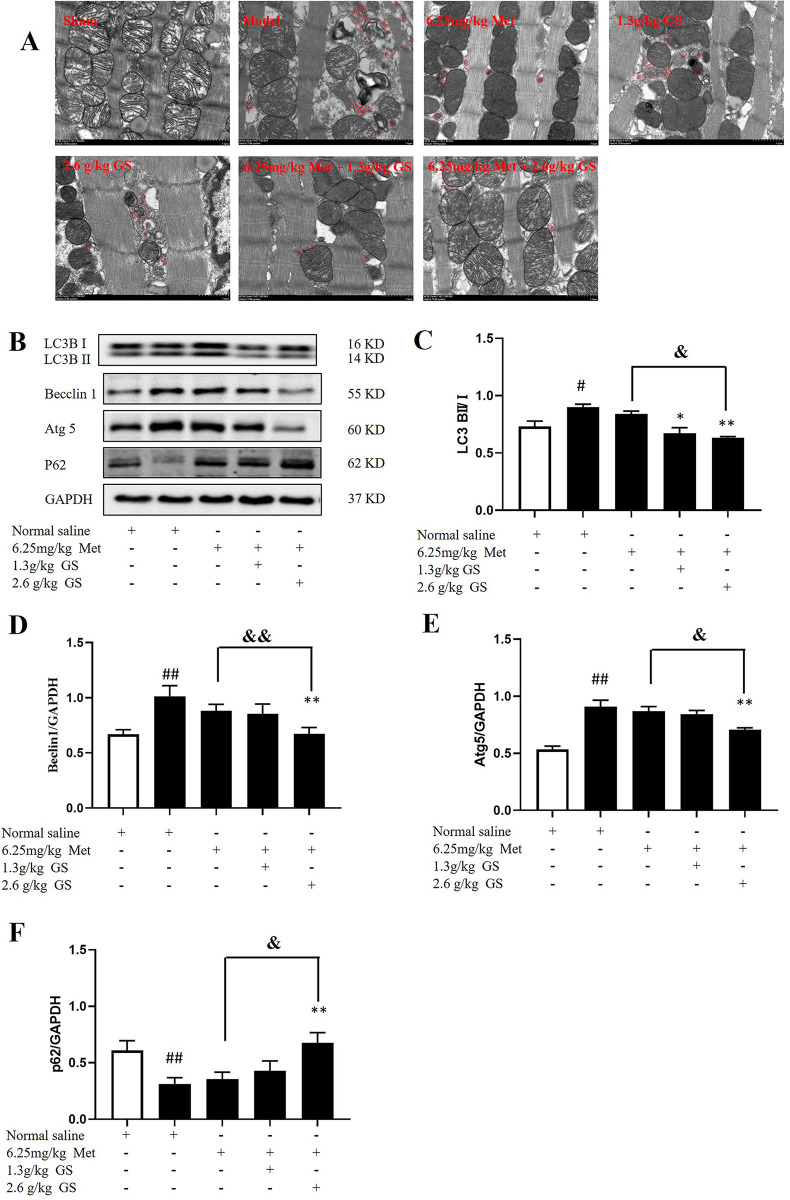
Effects of GS combined with metoprolol (Met) on autophagy-related proteins in heart failure mice. (A) Typical transmission electron microscopy image. (B-F) Western blot analysis of autophagy-related proteins (LC3BII/I, p62, Beclin1, and Atg5). Data are represented as mean ± SEM (n = 6). # p < 0.05 or ## p < 0.01 vs. sham. * p < 0.05 or ** p < 0.01 vs. model. & p < 0.05 or && p < 0.01 vs. 2.6g/kg GS or 6.25mg/kg Met.

Transmission electron microscopy revealed that LAD ligation-induced HF increased autophagosomes in cardiomyocytes and induced mitochondrial swelling and disorganization compared with sham-operated mice. Met reduced autophagosomes in cardiomyocytes and improved mitochondrial swelling and derangement compared with the model mice. GS in 1.3 g/kg and 2.6 g/kg reduced autophagosomes; however, no change in mitochondrial swelling and disorganization was observed compared with the model mice. GS at 2.6 g/kg further reduced autophagosomes and improved mitochondrial swelling and derangement compared with Met alone (**Fig 5A**).

Further, we detected autophagy-related proteins. Compared with the sham group, the protein expression levels of LC3II/I, Beclin1, and Atg5 in the model group were significantly increased (p < 0.05 or p < 0.01). In contrast, the expression level of p62 was significantly decreased (p < 0.01). Compared with the model group, the protein expression levels of LC3bII/I, Beclin1, and Atg5 in the 6.25 mg/kg Met + 2.6 g/kg GS group were significantly decreased (p < 0.01); in contrast, the expression level of p62 was significantly increased (p < 0.05). Compared with the 6.25 mg/kg Met group, the protein expression levels of LC3bII/I, Beclin1, and Atg5 in the 6.25 mg/kg Met + 2.6 g/kg GS group were significantly decreased (p < 0.05 or p < 0.01); in contrast, the expression level of p62 was significantly increased (p < 0.05, **Fig 5B–5F**).

### Effects of GS combined with Met on PI3K, p-PI3K, p-Akt, Akt, p-mTOR, and mTOR in the upstream signaling pathway of autophagy in mice with HF

**Fig 6:**
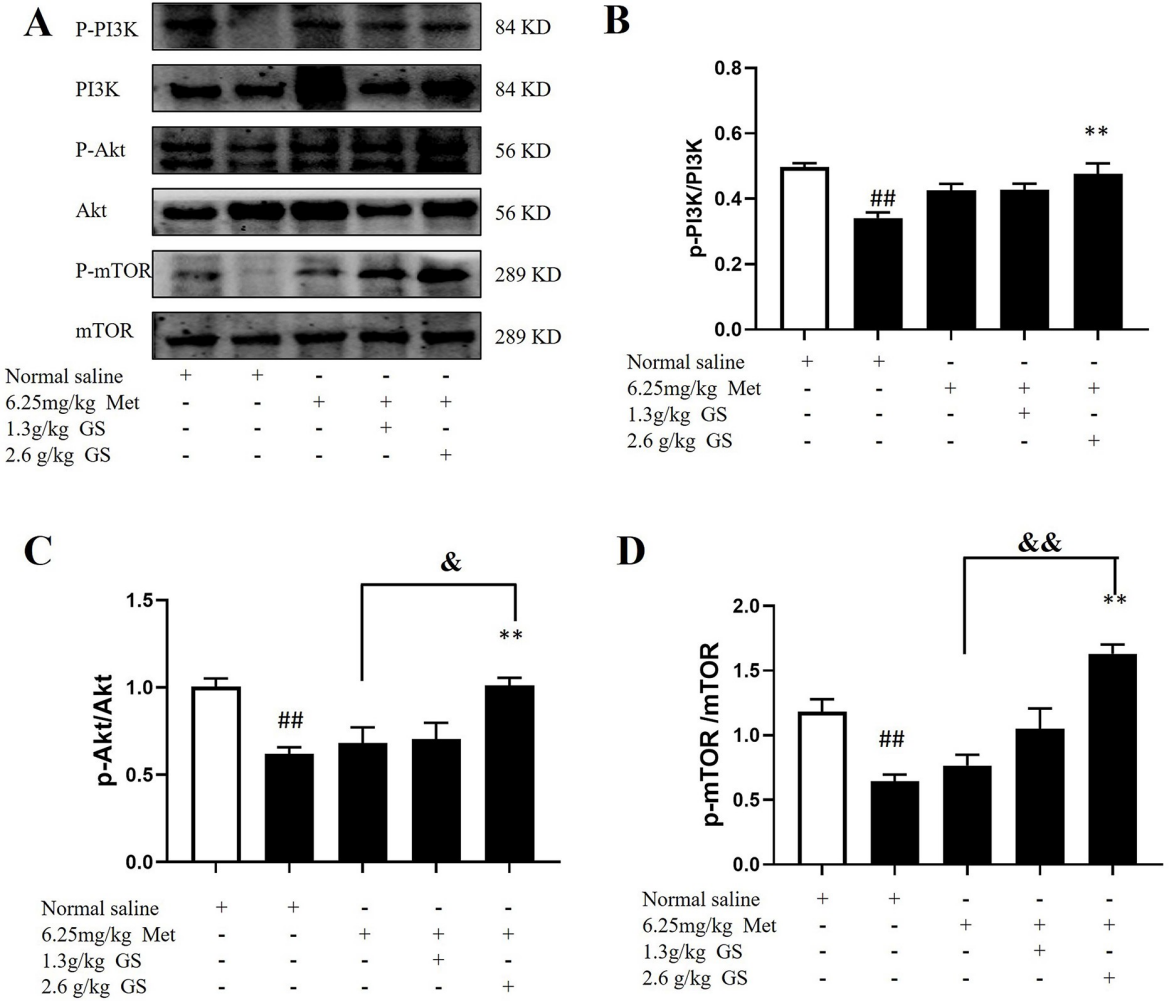
Effects of GS combined with Met on PI3K/Akt/mTOR pathway in heart failure mice. (A) Typical bands of western blot. (B-D) Western blot analysis of p-PI3K/PI3K, p-Akt/Akt, and p-mTOR/mTOR. Data are represented as mean ± SEM (n = 6). ## p < 0.01 vs. sham. * p < 0.05 or ** p < 0.01 vs. model. & p < 0.05 or && p < 0.01 vs. 2.6g/kg GS or 6.25mg/kg Met.

Western blot results for p-PI3K/PI3K, p-Akt/Akt, and p-mTOR/mTOR are shown in **Fig 6A**. The ratios of p-PI3K/PI3K, p-Akt/Akt, and p-mTOR/mTOR were decreased in the model group compared with the sham group (p < 0.01), and they were higher in the 6.25 mg/kg Met + 2.6 g/kg GS group than in the model group (p < 0.01). There were no significant differences between the other groups and the model group. The p-Akt/Akt and p-mTOR/mTOR ratios were significantly higher in the 6.25 mg/kg Met + 2.6 g/kg GS group than in the Met group (p < 0.01) (**Fig 6B**), while the p-PI3K/PI3K ratio was not significantly different (p >0.05, **Fig 6C and 6D**).

## Discussion

**Fig 7:**
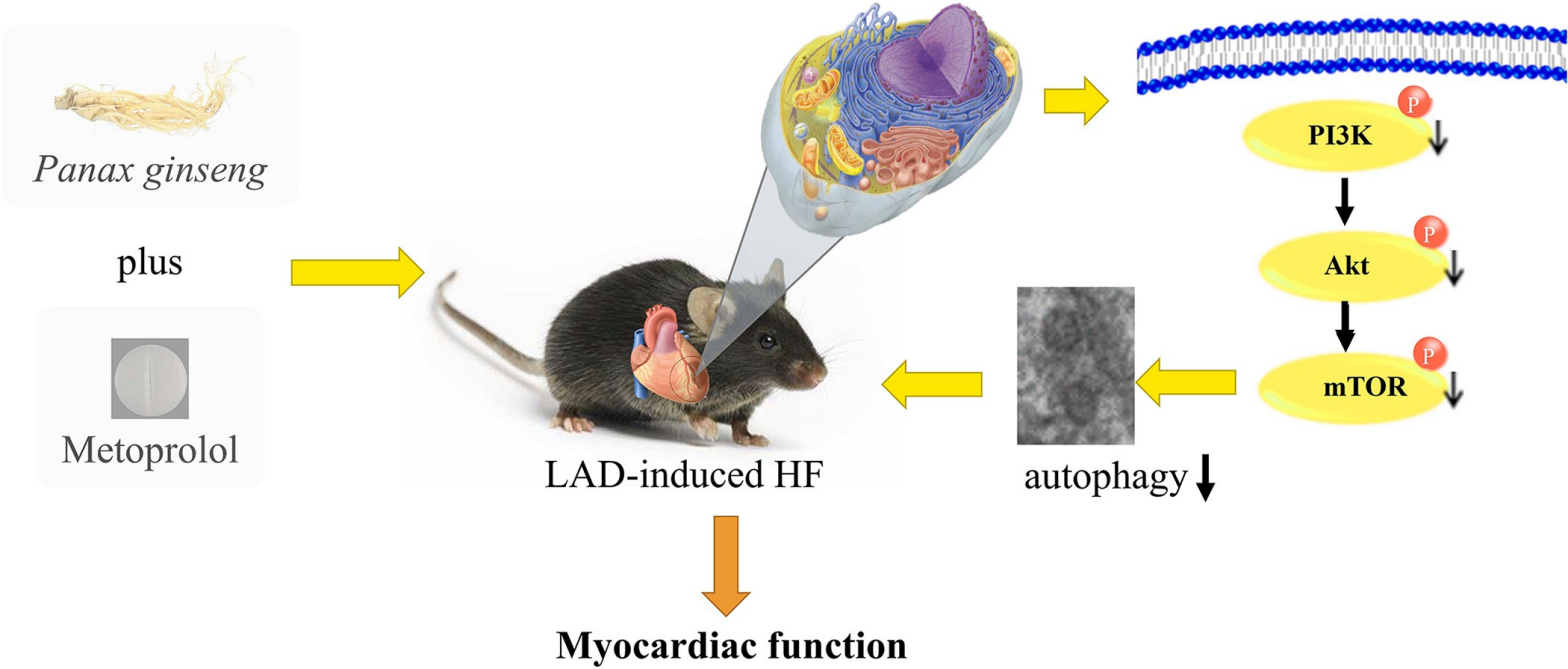
Summary graph of this study.

Heart failure is a significant public health challenge as it is the leading cause of death and disability worldwide. Heart failure is becoming more common despite great advancements achieved in heart disease treatment, and the survival rate has only slightly improved. Ginseng has been used extensively around the world for a long time. Numerous studies on animals have shown that Ginseng and ginsenosides were effective in treating cardiovascular diseases. Ginseng extract and ginsenosides regulate energy metabolism and nervous system function to alleviate heart failure in rats with HF [19, 20]. Ginsenosides also have protective effects on the heart, increasing the viability and ATP levels in H9c2 cells while reducing oxidative damage [21]. Ginsenoside Rg3 enhances cardiac function in heart failure by facilitating the SUMOylation of SERCA2a [22]. By regulating the TGF-β1/Smad3 pathway, ginsenoside alleviates isoproterenol-induced myocardial fibrosis and heart failure [23]. Recent research has also reported that Ginseng and ginsenosides modulate mitochondrial homeostasis in cardiovascular diseases [24, 25]. Ginsenoside Rg1 inhibits cardiac remodeling in heart failure through SIRT1/PINK1/Parkin-mediated mitochondrial autophagy. Ginsenoside Rd increases reticulin secretion in adipose tissue via TBK1-AMPK and enhances mitochondrial biogenesis in heart failure through the Wnt5a/Ca^2+^ pathway [26]. Small-scale clinical trials have demonstrated the potential benefits of traditional Chinese medicine containing ginseng such as Shenmai San used as an adjuvant therapy for patients with heart failure [27, 28]. The use of Ginseng in conjunction with conventional Western therapy to treat heart failure is still unproven, nevertheless. Consequently, GS and metoprolol were administered in combination in this study in male C57BL/6J mice with post-LAD ligation-induced heart failure. It was discovered that a combination of 6.25 mg/kg Met and GS significantly reduced the decline in cardiac function in these animals (Fig 7).

Nearly all eukaryotic cells exhibit autophagy, which is a normal biological process. By producing autophagosomes and lysosomes, autophagy eliminates damaged or long-lived organelles, protein aggregates, and undesirable cell complexes. Excessive or uncontrolled autophagy induction may be detrimental and maladaptive, aggravating the pathophysiology of heart failure (HF), even while modest to moderate autophagy induction is thought to be cell-protective and adaptive [29, 30]. That means that autophagy in cardiac tissue is a double-edged sword. Following translation, the microtubule-associated protein LC3 is altered and transformed into LC3-II. It signals the development of autophagy by covalently binding to phosphatidylethanolamine (PE). An increase in autophagy is indicated by a rise in the ratio of LC3-II to LC3-I [31]. The decline of the p62 protein, an autophagy substrate, means an increase in autophagy. Atg5 combines with Atg12 and Atg16 to generate complexes that facilitate autophagosome membrane expansion and maturation of autophagosome. According to the results from our study, the combination of Met with GS significantly prevented the increase in the ratio of LC3-II to LC3-I in heart failure mice, reduced the expression of the autophagy markers Beclin 1 and Atg5, and reversed the decrease in the p62 protein. This suggests that GS, along with Met, may have improved cardiac function in heart failure mice by preventing myocardial damage caused by excessive autophagy.

AMPK, ROS signaling pathway, and mTOR are signaling pathways that control autophagy. It is reported that mTOR is a sensor for hormones, ATP, and amino acids. It serves as a negative regulator of autophagy activity. When there is a shortage of cellular energy, adenosine-activated protein kinase (AMPK) is activated, which inhibits mTOR and promotes the initiation of autophagy [32]. Mitochondrial transplantation reduces cardiac cell death by blocking excessive autophagy mediated by AMPK-mTOR [33]. The mTOR pathway is also important in heart failure (HF). It has been demonstrated that HF may be treated and prevented by inhibiting mTOR-related autophagy [34]. Specialists have made progress in the regulatory mechanisms of traditional Chinese medicine on autophagy during heart failure. By controlling autophagy, traditional Chinese medicine may be able to slow the progression of HF. The Shenfu Injection, which contains GS, can improve heart function by inhibiting autophagy and activating the PI3K/Akt/mTOR signaling pathway [35]. Tetrahydrocurcumin activates the PI3K/Akt/mTOR signaling pathway to prevent hypoxia/reoxygenation-induced autophagy [36]. Typhaneoside enhances the expression of p62 protein, increases the p-Akt/Akt and p-mTOR/mTOR ratios, and significantly reduces the number of autophagosomes and the LC3-II/LC3-I ratio in HF rats, which means that typhaneoside reduces autophagy and enhances cardiac function through the PI3K/Akt/mTOR signaling pathway [37]. The results of our present study align with these mentioned observations above, indicating that the cardioprotective effect of Met combination therapy with GS may be mediated by inhibiting autophagy via the PI3K/Akt/mTOR signaling pathway.

### Clinical implications

Although beta blockers are the preferred medication to protect the heart from compensatory hypersensitivity, the widespread use of beta receptor blockers has also improved the survival rate of patients with heart failure. However, many patients still face the risk of death caused by heart failure. This study suggests that in mice suffering LAD ligation-induced HF, the combination of metoprolol and GS treatment can better exert cardioprotective effects than metoprolol alone. GS can exert a coordinated anti-heart failure effect with metoprolol through the PI3K/Akt/mTOR pathway-mediated autophagy inhibition. Our research suggests that combining metoprolol with GS in patients with chronic heart failure caused by myocardial infarction may provide clinical benefits.

### Limitations

The pathological process of heart failure is very complex, including systolic and diastolic dysfunction, pressure or volume overload, hemodynamic instability, maladaptive neurohumoral activation, and adverse ventricular remodeling [38]. Various cellular and molecular mechanisms lead to severe myocardial cell apoptosis, chronic low-grade inflammation, myocardial fibrosis, calcium cycling disorders or overload in myocardial cells, mitochondrial dysfunction, and excessive oxidative stress. Hence, one of the limitations of this study is that the cardioprotective effect of combination therapy (GS plus Met) was only examined in the HF model induced by LAD ligation. The results of this work are encouraging and worth further exploration in other heart failure models in the future. In addition, more and more investigations paid attention to the adverse cardiovascular events of adjuvant Chinese patent medicine therapy [39]. The present study only focused on the effect of GS combined with metoprolol on cardiac function. Further work on other potential adverse effects of combination therapy is needed in the short and long term to gain a better understanding of the risks and benefits.

## Conclusion

We demonstrated that a combination of GS with metoprolol improved heart function in HF mice by inhibiting autophagy through the PI3K/Akt/mTOR signaling pathway.

## Supporting information

S1 Raw data (XLSX)

S1 Raw image (PDF)
