# Supplementary material for: Ginseng Radix et Rhizoma enhanced the effect of metoprolol in chronic heart failure by inhibiting autophagy in male C57BL/6J mice
Source: PLoS One. 2024 Aug 14;19(8):e0301875. doi: 10.1371/journal.pone.0301875 (PMC11324128; doi:10.1371/journal.pone.0301875)

Figure 5b Atg5

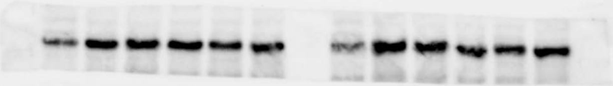

Figure 5b beclin 1

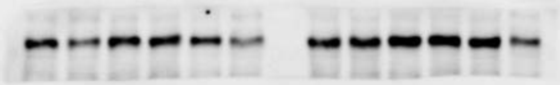

Figure 5b GAPDH

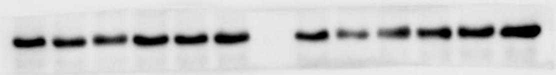

Figure 5b LC3

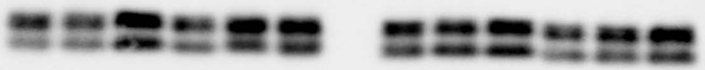

Figure 5b p62

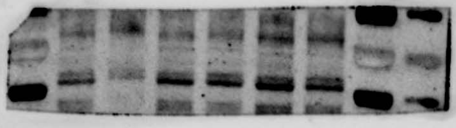

Figure 6A Akt

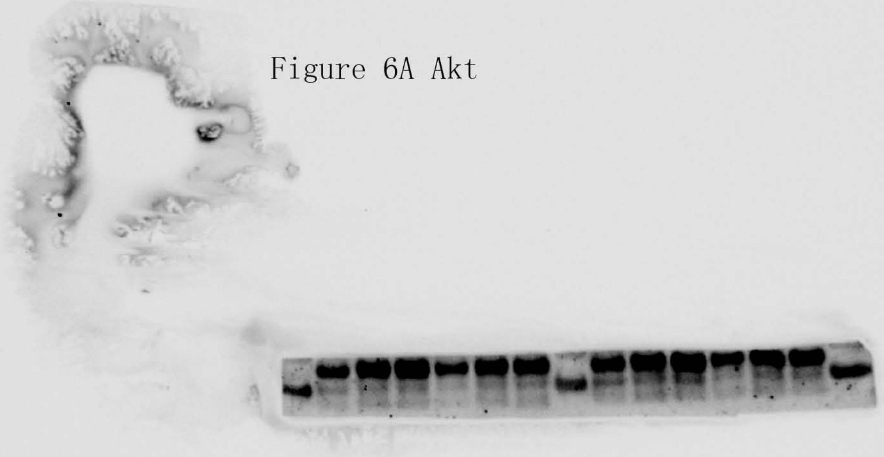

Figure 6A mTOR

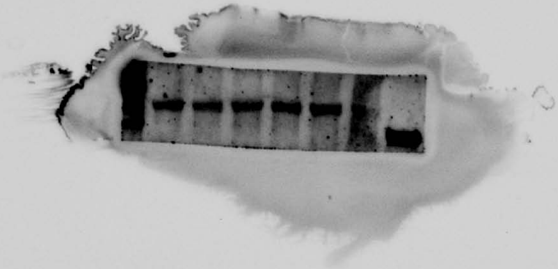

Figure 6A p-Akt

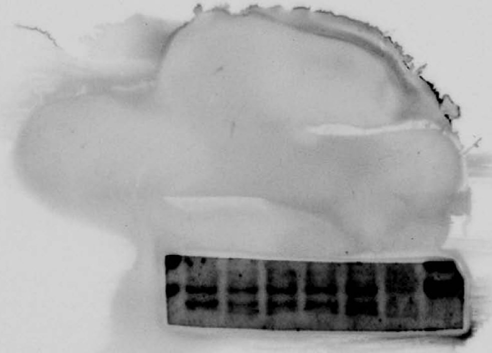

Figure 6A PI3K

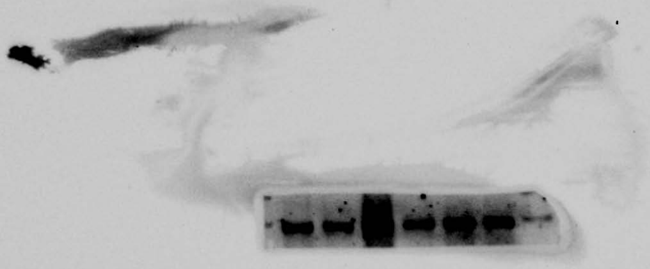

Figure 6A

P-mTOR

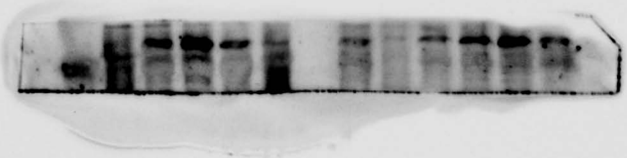

Figure 6A p-PI3K

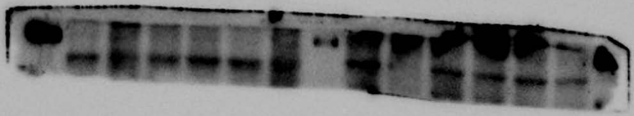

Supplement: S1 Raw image — (PDF) [file pone.0301875.s002.pdf]
